# Fibrinogen-to-red blood cell ratio is associated with disease burden and short-term outcomes in hospitalized patients with gout: comparison with conventional inflammatory indices

**DOI:** 10.3389/fmed.2026.1879555

**Published:** 2026-07-15

**Authors:** Hao-Jie Zhong, Shu-Juan Chen, Xiao-Lan Qian, Min Xiao, Gui-Chen Ling, Jian-Yong Zhang, Jing-Jing Xie

**Affiliations:** Department of Rheumatology, Shenzhen Traditional Chinese Medicine Hospital, The Fourth Clinical Medical College of Guangzhou University of Chinese Medicine, Shenzhen, China

**Keywords:** erythrocytes, fibrinogen, gout, prognosis, proteinuria

## Abstract

**Background:**

The fibrinogen-to-red blood cell ratio (FRR) has not been evaluated in gout. This study investigated the association of FRR with disease burden, inflammatory status, renal involvement, and short-term prognosis in hospitalized patients with gout, and compared its performance with the neutrophil-to-lymphocyte ratio (NLR) and platelet-to-lymphocyte ratio (PLR).

**Methods:**

This single-center retrospective study included 330 consecutive hospitalized patients with gout admitted between January 2024 and August 2025. Patients were stratified into low- and high-FRR groups according to the median FRR. Clinical characteristics, inflammatory markers, renal indicators, and hospitalization outcomes were compared between groups. Correlation analyses, multivariable linear and logistic regression models, and receiver operating characteristic (ROC) analyses were performed.

**Results:**

Compared with the low-FRR group, patients with high FRR had more frequent annual flares, a higher prevalence of tophi, more affected joints, higher levels of CRP and ESR, higher white blood cell, neutrophil, and monocyte counts, more frequent proteinuria, longer hospital stay, higher hospitalization costs, and higher 30-day readmission rates (all *P* < 0.05). FRR showed stronger correlations with CRP and ESR than NLR and PLR. After adjustment, higher FRR remained independently associated with annual flare frequency and systemic inflammatory markers. High FRR was also independently associated with tophi, proteinuria, and 30-day readmission. FRR showed higher AUCs than NLR and PLR for tophi, proteinuria, and 30-day readmission.

**Conclusion:**

FRR may serve as a simple adjunctive biomarker associated with disease burden, inflammatory activity, and selected short-term hospitalization outcomes in hospitalized patients with gout.

## Graphical Abstract

**Figure:**
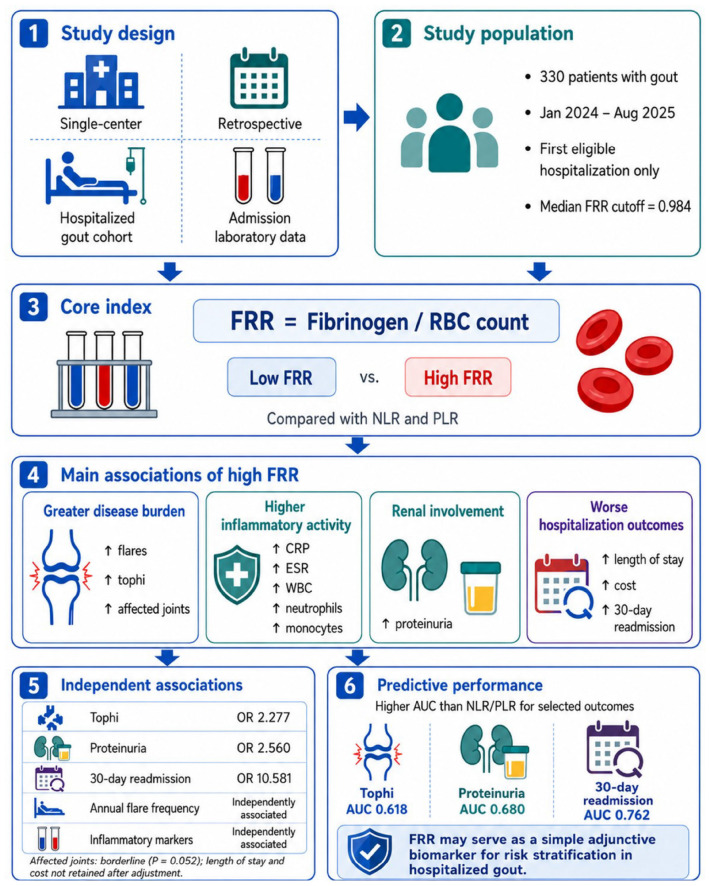
FRR was calculated from routine admission laboratory data in 330 hospitalized patients with gout and compared with NLR and PLR. High FRR was associated with greater disease burden, higher inflammatory activity, proteinuria, and worse short-term hospitalization outcomes. After adjustment, FRR remained independently associated with annual flare frequency, inflammatory markers, tophi, proteinuria, and 30-day readmission. These findings support the potential value of FRR as a simple adjunctive biomarker for risk stratification in hospitalized gout.

## 1 Introduction

Gout is among the most common chronic inflammatory arthritis diseases worldwide, with a global prevalence of approximately 1–4% and a steadily increasing disease burden (1–3). Clinically, persistent or poorly controlled gout can lead to recurrent flares, tophaceous deposition, progressive joint damage, and substantial comorbidity burden, resulting in considerable disability and healthcare utilization (3, 4).

Currently, several inflammatory markers, including C-reactive protein (CRP), erythrocyte sedimentation rate (ESR), and composite hematologic indices such as the neutrophil-to-lymphocyte ratio (NLR) and platelet-to-lymphocyte ratio (PLR), are widely used in clinical practice to evaluate disease activity in gout (5–7). Accumulating evidence suggests that both NLR and PLR are elevated in gout, especially during acute attacks, and correlate with inflammatory activity (5, 8, 9). Nevertheless, these markers reflect only limited aspects of the inflammatory response. Therefore, more comprehensive and reliable indicators are still needed to better characterize the overall inflammatory status and disease burden in patients with gout.

Fibrinogen is a key acute-phase reactant involved in both inflammation and coagulation, and limited evidence suggests that elevated fibrinogen levels may be associated with disease activity in gout (10). In parallel, red blood cell (RBC) count and RBC-related alterations are closely linked to systemic inflammatory states and have been associated with disease activity, comorbidity burden, and prognosis in chronic inflammatory disorders (11–13). In addition, fibrinogen is a major determinant of RBC aggregation and may influence blood rheology and microcirculatory perfusion (14, 15). Taken together, these findings provide a biological rationale for integrating fibrinogen and RBC-related parameters into a composite index, which may offer a more comprehensive assessment of inflammatory burden and prognostic risk.

To date, the fibrinogen-to-red blood cell ratio (FRR) has not been specifically evaluated in gout. Accordingly, the present study investigated the association of FRR with disease burden, inflammatory status, proteinuria-related renal involvement, and short-term hospitalization outcomes in patients with gout, and compared its predictive performance with conventional inflammatory indices, including NLR and PLR, to explore its potential clinical utility.

## 2 Materials and methods

### 2.1 Study design and participants

This single-center retrospective study enrolled consecutive patients with gout who were hospitalized in the Department of Rheumatology, Shenzhen Traditional Chinese Medicine Hospital, between January 2024 and August 2025. Patients were eligible if they had a documented diagnosis of gout based on the 2015 American College of Rheumatology/European League Against Rheumatism (ACR/EULAR) gout classification criteria (16) in the electronic medical record. Eligible patients were identified through the electronic medical record system. All consecutive eligible hospitalizations during the predefined study period were screened for inclusion. Patients were excluded if admission fibrinogen or RBC count was unavailable, key clinical or laboratory data were incomplete, or the hospitalization represented a duplicate admission from the same patient. No additional disease-based exclusion criteria were applied. Conditions that may affect fibrinogen or RBC levels, such as active infection, hematologic disorders, malignancy, or other acute inflammatory conditions, could not be uniformly identified in this retrospective dataset and were therefore addressed as a limitation. Because this was an exploratory retrospective study, no formal sample size calculation was performed. For patients with multiple admissions during the study period, only the first eligible hospitalization was retained for analysis. To reduce selection bias, consecutive case screening was performed through the same institutional electronic medical record system, and duplicate admissions from the same patient were excluded from repeated inclusion.

### 2.2 Data collection

Demographic, clinical, laboratory, hospitalization-related, and medication data were extracted from inpatient electronic medical records. Demographic and clinical variables included age, sex, smoking status, alcoholism, hypertension, type 2 diabetes, serum uric acid level, disease duration, annual flare frequency, presence of tophi, number of affected joints, joint swelling, and functional limitation. Admission laboratory parameters included plasma fibrinogen, RBC count, CRP, ESR, white blood cell count, neutrophil count, monocyte count, lymphocyte count, serum creatinine, estimated glomerular filtration rate (eGFR), urinary red blood cell count, and proteinuria. Hospitalization-related outcomes included length of hospital stay, hospitalization cost, 30-day readmission, in-hospital venous thrombosis, and in-hospital mortality. Medication use at admission, including corticosteroids, colchicine, nonsteroidal anti-inflammatory drugs, and urate-lowering therapy, was also recorded. To reduce information bias, all variables were extracted from the same electronic medical record system according to prespecified definitions, and laboratory indicators used for index calculation were based on admission data. Proteinuria was defined according to the admission urinalysis result and was recorded as present when urinary protein was reported as positive.

### 2.3 Calculation of FRR, NLR, and PLR

Plasma fibrinogen was measured in g/L, and RBC count was expressed as × 10^12^/L. FRR was calculated as plasma fibrinogen concentration divided by RBC count and was expressed as g/L per 10^12^/L. NLR and PLR were calculated as the absolute neutrophil count and platelet count divided by the absolute lymphocyte count, respectively. To evaluate the clinical significance of FRR, patients were stratified into low-FRR and high-FRR groups according to the median FRR value of the study population.

### 2.4 Study outcomes

The main objective of this study was to investigate the association of FRR with disease burden, inflammatory status, renal involvement, and short-term hospitalization outcomes in patients with gout. Indicators of disease burden included disease duration, annual flare frequency, presence of tophi, number of affected joints, joint swelling, and functional limitation. Inflammatory status was assessed using CRP, ESR, and peripheral blood cell counts. Renal involvement was evaluated using serum creatinine, eGFR, urinary red blood cell count, and proteinuria. Hospitalization outcomes included length of hospital stay, hospitalization cost, 30-day readmission, in-hospital venous thrombosis, and in-hospital mortality. For reporting purposes, disease burden and inflammatory indicators were considered the main clinical outcomes, whereas renal involvement and short-term hospitalization outcomes were treated as secondary outcome domains.

### 2.5 Statistical analysis

All statistical analyses were performed using GraphPad Prism version 8.0 (GraphPad Software, San Diego, CA, United States) and IBM SPSS Statistics version 25.0 (IBM Corp., Armonk, NY, United States). The distribution of continuous variables was assessed using the Shapiro–Wilk test. Normally distributed continuous variables are presented as mean ± standard deviation (SD) and were compared using the independent-samples Student’s *t*-test, whereas non-normally distributed continuous variables are presented as median (interquartile range [IQR]) and were compared using the Mann–Whitney *U* test. Categorical variables are expressed as number (percentage) and were compared using the chi-square test or Fisher’s exact test, as appropriate.

Correlations of FRR, NLR, and PLR with clinical characteristics, inflammatory markers, renal function indices, and hospitalization outcomes were assessed using Pearson’s correlation for normally distributed continuous variables and Spearman’s rank correlation for skewed or ordinal variables, as appropriate. Separate multivariable linear regression models were constructed using a stepwise selection procedure to evaluate the associations between FRR group (high vs. low, treated as a categorical variable) and selected continuous outcomes. Separate multivariable logistic regression models were constructed using a forward selection procedure, with FRR group entered as the main candidate independent variable in the variable selection procedure, to evaluate selected binary outcomes including tophi, proteinuria, and 30-day readmission. Candidate covariates included age, sex, smoking status, alcoholism, hypertension, type 2 diabetes, serum uric acid, corticosteroid use at admission, colchicine use at admission, NSAID use at admission, and urate-lowering therapy at admission. Skewed continuous outcomes were log-transformed when appropriate before multivariable linear regression analyses, and model assumptions were checked. Results of linear regression analyses are presented as regression coefficients (β) with standard errors (SEs), and results of logistic regression analyses are presented as odds ratios (ORs) with 95% confidence intervals (CIs). Given the very small number of in-hospital venous thrombosis events and the absence of in-hospital deaths, adjusted regression analyses were not performed for these outcomes.

Receiver operating characteristic (ROC) curve analysis was used to compare the predictive performance of FRR, NLR, and PLR for disease severity and short-term prognosis, including tophi, proteinuria, and 30-day readmission. Predictive ability was assessed by the area under the ROC curve (AUC) with corresponding 95% CIs. Optimal cutoff values were determined by maximizing the Youden index. All statistical tests were two-sided, and a *P* < 0.05 was considered statistically significant. Analyses involving variables with missing data were performed on an available-case basis.

### 2.6 Ethics statement

This study was approved by the Ethics Committee of Shenzhen Traditional Chinese Medicine Hospital. Given the retrospective design and the use of anonymized clinical data, the requirement for informed consent was waived by the ethics committee (Approval No. K2026-057-01). The study was conducted in accordance with the principles of the Declaration of Helsinki.

## 3 Results

### 3.1 Baseline characteristics of the study population

A total of 330 hospitalized patients with gout were included in the final analysis and were stratified into low-FRR and high-FRR groups according to the median FRR value (0.984). Overall, the study population was predominantly male. Compared with the low-FRR group, patients in the high-FRR group were older (*P* = 0.015; Table 1). Sex distribution, smoking status, alcoholism, hypertension, type 2 diabetes, colchicine use, NSAID use, and serum uric acid levels were comparable between the two groups. However, corticosteroid use at admission was more frequent in the high-FRR group, whereas urate-lowering therapy use at admission was less frequent. These variables were therefore included as candidate covariates in the multivariable analyses.

**TABLE 1:** Baseline demographic and clinical characteristics of patients stratified by FRR.

Variable	Overall (*n* = 330)	Low FRR (*n* = 165)	High FRR (*n* = 165)	*P*-value
Age (years)	46.00 (36.00–59.00)	44.00 (35.00–58.00)	49.00 (38.50–60.00)	**0.015**
Male sex, *n* (%)	320 (96.97)	162 (98.18)	158 (95.76)	0.199
Smoking, *n* (%)	50 (15.15)	25 (15.15)	25 (15.15)	1.000
Alcoholism, *n* (%)	34 (10.30)	15 (9.09)	19 (11.52)	0.469
Hypertension, *n* (%)	98 (29.70)	49 (29.70)	49 (29.70)	1.000
Type 2 diabetes, *n* (%)	45 (13.64)	23 (13.94)	22 (13.33)	0.873
Corticosteroid use at admission, *n* (%)	149 (45.15)	48 (29.09)	101 (61.21)	**< 0.001**
Colchicine use at admission, *n* (%)	48 (14.55)	26 (15.76)	22 (13.33)	0.532
NSAID use at admission, *n* (%)	174 (52.73)	83 (50.30)	91 (55.15)	0.378
Urate-lowering therapy at admission, *n* (%)	253 (76.67)	138 (83.64)	115 (69.70)	**0.003**
Serum uric acid (μmol/L)	489.00 (414.00–577.25)	489.00 (416.50–581.50)	489.00 (411.00–567.00)	0.440

Data are presented as median (interquartile range) or number (percentage). FRR, fibrinogen-to-red blood cell ratio; NSAIDs, nonsteroidal anti-inflammatory drugs. Bold values indicate statistically significant *P* values (*P* < 0.05).

### 3.2 Association of FRR with gout disease burden

Patients with higher FRR showed a greater disease burden (Table 2). Specifically, the high-FRR group had more frequent annual gout flares (*P* = 0.020), a higher prevalence of tophi (*P* = 0.003), and a greater number of affected joints (*P* = 0.008) than the low-FRR group. Disease duration tended to be longer in the high-FRR group, although the difference did not reach statistical significance (*P* = 0.069). In contrast, joint swelling and functional limitation were similarly common in both groups (both *P* > 0.05). Overall, elevated FRR appeared to be more closely associated with cumulative disease burden and structural severity than with highly prevalent acute manifestations.

**TABLE 2:** Comparison of gout disease characteristics between the low-FRR and high-FRR groups.

Variable	Overall (*n* = 330)	Low FRR (*n* = 165)	High FRR (*n* = 165)	*P*-value
Disease duration (months)	98.00 (50.75–153.00)	89.00 (40.50–142.50)	105.00 (62.50–157.50)	0.069
Annual flare frequency	4.00 (3.00–7.00)	4.00 (3.00–6.00)	5.00 (3.00–7.50)	**0.020**
Tophi, *n* (%)	238 (72.12)	107 (64.85)	131 (79.39)	**0.003**
Number of affected joints	2.00 (1.00–3.00)	2.00 (1.00–2.50)	2.00 (1.00–3.00)	**0.008**
Joint swelling, *n* (%)	320 (96.97)	158 (95.76)	162 (98.18)	0.199
Functional limitation, *n* (%)	312 (94.55)	153 (92.73)	159 (96.36)	0.146

Data are presented as median (interquartile range) or number (percentage). FRR, fibrinogen-to-red blood cell ratio. Bold values indicate statistically significant *P* values (*P* < 0.05).

### 3.3 Association of FRR with inflammatory status

A clear gradient in systemic inflammatory activity was observed across FRR groups (Table 3). Compared with the low-FRR group, patients with high FRR had markedly higher CRP and ESR levels (both *P* < 0.001). In parallel, white blood cell count, neutrophil count, and monocyte count were significantly increased (all *P* < 0.01), whereas lymphocyte count was significantly lower (*P* = 0.009). These findings indicate that elevated FRR is accompanied by a more pronounced inflammatory phenotype in patients with gout.

**TABLE 3:** Comparison of inflammatory markers between the low-FRR and high-FRR groups.

Variable	Overall (*n* = 330)	Low FRR (*n* = 165)	High FRR (*n* = 165)	*P*-value
C-reactive protein (mg/L)	16.50 (6.50–42.28)	8.30 (3.70–16.85)	34.20 (16.35–71.90)	**< 0.001**
Erythrocyte sedimentation rate (mm/h)	25.50 (11.00–57.00)	12.00 (5.00–20.50)	55.00 (31.00–76.00)	**< 0.001**
White blood cell count (10^9^/L)	7.90 (6.28–10.32)	7.04 (5.76–9.03)	8.81 (7.15–11.47)	**< 0.001**
Neutrophil count (10^9^/L)	4.76 (3.46–7.02)	3.99 (3.19–5.42)	5.83 (4.01–8.22)	**< 0.001**
Monocyte count (10^9^/L)	0.63 (0.45–0.82)	0.57 (0.42–0.75)	0.65 (0.50–0.88)	**0.007**
Lymphocyte count (10^9^/L)	1.98 (1.56–2.57)	2.10 (1.68–2.66)	1.85 (1.50–2.47)	**0.009**

Data are presented as median (interquartile range) or number (percentage). FRR, fibrinogen-to-red blood cell ratio. Bold values indicate statistically significant *P* values (*P* < 0.05).

### 3.4 Association of FRR with renal involvement

With respect to renal-related indicators, serum creatinine and eGFR did not differ significantly between groups (both *P* > 0.05), and urinary red blood cell count showed only a borderline trend toward higher levels in the high-FRR group (*P* = 0.064) (Table 4). However, proteinuria was significantly more frequent among patients with elevated FRR (*P* = 0.014). These results suggest that FRR may be more closely related to clinically apparent renal involvement than to conventional renal filtration indices alone.

**TABLE 4:** Comparison of renal function indicators between the low-FRR and high-FRR groups.

Variable	Overall (*n* = 330)	Low FRR (*n* = 165)	High FRR (*n* = 165)	*P*-value
Serum creatinine (μmol/L)	94.00 (82.00–110.00)	95.00 (82.00–108.00)	91.00 (82.00–115.00)	0.772
Estimated glomerular filtration rate	84.50 (70.00–96.00)	85.00 (71.00–98.00)	84.00 (68.00–95.00)	0.282
Urinary red blood cell count (RBCs/HPF)	1.00 (0.00–4.00) (*n* = 328)	0.00 (0.00–2.50) (*n* = 165)	1.00 (0.00–5.00) (*n* = 163)	0.064
Proteinuria, *n* (%)	26 (7.88)	7 (4.24)	19 (11.52)	**0.014**

Data are presented as median (interquartile range) or number (percentage). FRR, fibrinogen-to-red blood cell ratio; RBCs/HPF, red blood cells per high-power field. Bold values indicate statistically significant *P* values (*P* < 0.05).

### 3.5 Association of FRR with hospitalization outcomes

Patients in the high-FRR group had less favorable short-term hospitalization outcomes (Table 5). They experienced a longer hospital stay (*P* < 0.001), higher hospitalization costs (*P* = 0.008), and a higher rate of 30-day readmission (*P* = 0.006) compared with those in the low-FRR group. In-hospital venous thrombosis was rare and did not differ significantly between groups (*P* = 0.478), and no in-hospital deaths occurred. Together, these findings suggest that higher FRR is associated not only with greater disease severity, but also with increased healthcare utilization and a poorer short-term clinical course.

**TABLE 5:** Comparison of hospitalization outcomes between the low-FRR and high-FRR groups.

Variable	Overall (*n* = 330)	Low FRR (*n* = 165)	High FRR (*n* = 165)	*P*-value
Length of hospital stay (days)	7.00 (5.00–9.00)	6.00 (4.00–8.00)	7.00 (6.00–10.00)	**< 0.001**
Hospitalization cost (¥)	9279.04 (6579.20–11981.12)	8658.15 (6269.04–11085.76)	9728.02 (7105.90–13165.62)	**0.008**
30-day readmission, *n* (%)	11 (3.33)	1 (0.61)	10 (6.06)	**0.006**
In-hospital venous thrombosis, *n* (%)	2 (0.61)	0 (0)	2 (1.21)	0.478
Mortality, *n* (%)	0 (0)	0 (0)	0 (0)	NA

Data are presented as median (interquartile range) or number (percentage). FRR, fibrinogen-to-red blood cell ratio. P values were not calculated for outcomes with no events. Bold values indicate statistically significant *P* values (*P* < 0.05).

### 3.6 Correlations of FRR, NLR, and PLR with clinical indicators

Correlation analyses further supported the clinical relevance of FRR (Table 6). FRR was positively correlated with disease duration (*r* = 0.129, *P* = 0.019), annual flare frequency (*r* = 0.140, *P* = 0.011), and number of affected joints (*r* = 0.157, *P* = 0.004). Notably, FRR showed strong positive correlations with CRP (*r* = 0.693, *P* < 0.001) and ESR (*r* = 0.794, *P* < 0.001), and these correlations were stronger than those observed for NLR and PLR. FRR was also significantly correlated with white blood cell count, neutrophil count, monocyte count, urinary red blood cell count, length of hospital stay, and hospitalization cost (all *P* < 0.01), whereas no significant correlation was found with serum creatinine or eGFR (both *P* > 0.05). Overall, compared with NLR and PLR, FRR demonstrated a broader and stronger pattern of associations with inflammatory and clinically relevant disease indicators.

**TABLE 6:** Correlations of FRR, NLR, and PLR with clinical characteristics and inflammatory markers in patients with gout.

Variable	FRR (r)	*P*	NLR (*r*)	*P*	PLR (*r*)	*P*
Disease duration (months)	0.129	**0.019**	0.029	0.595	0.041	0.453
Annual flare frequency	0.140	**0.011**	0.097	0.079	0.121	**0.028**
Number of affected joints	0.157	**0.004**	0.162	**0.003**	0.179	**0.001**
C-reactive protein (mg/L)	0.693	**< 0.001**	0.424	**< 0.001**	0.344	**< 0.001**
Erythrocyte sedimentation rate (mm/h)	0.794	**< 0.001**	0.317	**< 0.001**	0.365	**< 0.001**
White blood cell count (10^9^/L)	0.356	**< 0.001**	0.540	**< 0.001**	0.150	**0.001**
Neutrophil count (10^9^/L)	0.412	**< 0.001**	0.786	**< 0.001**	0.371	**< 0.001**
Monocyte count (10^9^/L)	0.178	**0.001**	0.239	**< 0.001**	-0.037	0.503
Serum creatinine (μmol/L)	0.038	0.491	0.102	0.064	0.074	0.179
Estimated glomerular filtration rate	-0.093	0.091	-0.049	0.376	-0.023	0.678
Urinary red blood cell count (RBCs/HPF)	0.162	**0.003**	0.089	0.107	0.063	0.255
Length of hospital stay (days)	0.246	**< 0.001**	0.216	**< 0.001**	0.187	**0.001**
Hospitalization cost (¥)	0.184	**< 0.001**	0.084	0.129	0.044	0.427

FRR, fibrinogen-to-red blood cell ratio; NLR, neutrophil-to-lymphocyte ratio; PLR, platelet-to-lymphocyte ratio; RBCs/HPF, red blood cells per high-power field. Bold values indicate statistically significant *P* values (*P* < 0.05).

### 3.7 Multivariable associations between FRR and disease-related indicators

After adjustment for candidate covariates, including demographic factors, comorbidities, serum uric acid, and medication use at admission, high FRR remained independently associated with several unfavorable disease-related indicators (Table 7). In multivariable linear regression analyses, high FRR was independently associated with greater annual flare frequency (*P* = 0.003) and higher levels of CRP (*P* < 0.001), ESR (*P* < 0.001), white blood cell count (*P* < 0.001), and neutrophil count (*P* < 0.001). The association with the number of affected joints showed only a borderline trend (*P* = 0.052). By contrast, FRR was not retained in the final multivariable models for disease duration, monocyte count, serum creatinine, eGFR, urinary red blood cell count, length of hospital stay, or hospitalization cost. These results suggest that the independent associations of FRR were mainly concentrated in flare burden and systemic inflammatory activity, whereas its associations with the number of affected joints, length of hospital stay, and hospitalization cost were attenuated after adjustment for major confounders and medication use at admission.

**TABLE 7:** Multivariable linear regression analyses of the associations between FRR and continuous disease-related indicators.

Variable	β	SE	*P*
Disease duration (months)	–	–	–
Annual flare frequency	0.978	0.323	**0.003**
Number of affected joints	0.324	0.166	0.052
C-reactive protein (mg/L)	40.131	4.422	**< 0.001**
Erythrocyte sedimentation rate (mm/h)	35.218	2.235	**< 0.001**
White blood cell count (10^9^/L)	1.672	0.334	**< 0.001**
Neutrophil count (10^9^/L)	1.902	0.291	**< 0.001**
Monocyte count (10^9^/L)	–	–	–
Serum creatinine (μmol/L)	–	–	–
Estimated glomerular filtration rate	–	–	–
Urinary red blood cell count (RBCs/HPF)	–	–	–
Length of hospital stay (days)	–	–	–
Hospitalization cost (¥)	–	–	–

FRR, fibrinogen-to-red blood cell ratio; SE, standard error. Candidate covariates included age, sex, smoking status, alcoholism, hypertension, type 2 diabetes, serum uric acid, corticosteroid use at admission, colchicine use at admission, NSAID use at admission, and urate-lowering therapy at admission. Separate multivariable linear regression models were fitted for each continuous outcome. –, FRR was not retained in the final multivariable model for that outcome. Bold values indicate statistically significant *P* values (*P* < 0.05).

In multivariable logistic regression analyses, high FRR was independently associated with increased risks of tophi (OR 2.277, 95% CI 1.369–3.786; *P* = 0.002), proteinuria (OR 2.560, 95% CI 1.004–6.527; *P* = 0.049), and 30-day readmission (OR 10.581, 95% CI 1.339–83.625; *P* = 0.025) (Table 8).

**TABLE 8:** Multivariable logistic regression analyses of the associations between FRR and binary clinical outcomes.

Variable	OR	95% CI	*P*
Tophi	2.277	1.369–3.786	**0.002**
Joint swelling	–	–	–
Functional limitation	–	–	–
Proteinuria	2.560	1.004–6.527	**0.049**
30-day readmission	10.581	1.339–83.625	**0.025**
In-hospital venous thrombosis	NA	NA	NA
Mortality	NA	NA	NA

FRR, fibrinogen-to-red blood cell ratio; OR, odds ratio; CI, confidence interval. Candidate covariates included age, sex, smoking status, alcoholism, hypertension, type 2 diabetes, serum uric acid, corticosteroid use at admission, colchicine use at admission, NSAID use at admission, and urate-lowering therapy at admission. Separate multivariable logistic regression models were fitted for each binary outcome. –, FRR was not retained in the final multivariable model or adjusted analysis was not performed because of sparse or absent events, as appropriate. NA, adjusted analysis was not performed because of sparse or absent events. Bold values indicate statistically significant *P* values (*P* < 0.05).

### 3.8 Predictive performance of FRR compared with NLR and PLR

**FIGURE 1:**
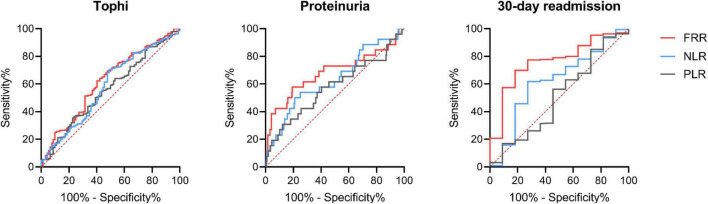
ROC curves comparing the predictive performance of FRR, NLR, and PLR for tophi, proteinuria, and 30-day readmission in hospitalized patients with gout. The selected outcomes included tophi, proteinuria, and 30-day readmission. AUC values with 95% confidence intervals are shown in Table 9.

ROC curve analyses indicated that FRR had potentially better discriminatory performance than NLR and PLR for tophi, proteinuria, and 30-day readmission (Figure 1 and Table 9). FRR yielded AUCs of 0.618 for tophi, 0.680 for proteinuria, and 0.762 for 30-day readmission, with optimal cutoffs of 0.905, 1.313, and 1.144, respectively. The corresponding sensitivities/specificities were 62.18%/59.78%, 57.69%/80.59%, and 81.82%/69.91%, respectively. Overall, these findings suggest that FRR may provide additional value over NLR and PLR for identifying patients with greater disease burden and increased short-term readmission risk.

**TABLE 9:** Comparison of predictive performance of FRR, NLR, and PLR for selected clinical outcomes.

Outcome	Indicator	AUC	95% CI	P	Optimal cutoff	Sensitivity (%)	Specificity (%)
Tophi	FRR	0.618	0.550–0.686	**0.001**	0.905	62.18	59.78
	NLR	0.562	0.494–0.630	0.083	3.190	36.55	76.09
	PLR	0.581	0.511–0.651	**0.023**	112.651	70.17	51.09
Proteinuria	FRR	0.680	0.547–0.813	**0.002**	1.313	57.69	80.59
	NLR	0.631	0.510–0.751	**0.027**	3.667	50.00	78.95
	PLR	0.576	0.446–0.706	0.200	227.333	30.77	87.83
30-day readmission	FRR	0.762	0.634–0.890	**0.003**	1.144	81.82	69.91
	NLR	0.619	0.444–0.794	0.180	2.797	72.73	61.76
	PLR	0.508	0.319–0.697	0.926	212.903	27.27	84.95

FRR, fibrinogen-to-red blood cell ratio; NLR, neutrophil-to-lymphocyte ratio; PLR, platelet-to-lymphocyte ratio. Bold values indicate statistically significant *P* values (*P* < 0.05).

## 4 Discussion

In this retrospective cohort of hospitalized patients with gout, higher FRR was associated with greater disease burden, stronger systemic inflammation, proteinuria, and poorer short-term outcomes. After multivariable adjustment, FRR remained independently associated with annual flare frequency, inflammatory markers, tophi, proteinuria, and 30-day readmission. These findings suggest that FRR mainly reflects inflammatory activity and selected adverse clinical outcomes, supporting its potential role as an adjunctive biomarker for risk stratification in hospitalized gout (Graphical abstract).

Our findings are broadly in line with prior work showing that fibrinogen is closely linked to inflammatory activity in gout. Chen et al. reported that plasma fibrinogen was elevated in active gout and could serve as a reliable marker for monitoring inflammatory response and disease activity (10). Likewise, previous studies have shown that NLR and PLR are elevated in gout and are associated with disease activity, while more recent multicenter data suggest that NLR is independently associated with inpatient gout recurrence (8). Beyond gout, serum-based inflammatory indices have also been linked to disease activity in other rheumatic diseases; for example, Atik and Atik reported that NLR, systemic immune-inflammation index, and uric acid-to-HDL cholesterol ratio were significantly associated with active disease in patients with ankylosing spondylitis (17). Against this background, our study extends the literature by showing that a composite index integrating fibrinogen and RBC count may outperform conventional hematologic inflammatory indices in capturing a wider spectrum of disease burden and short-term adverse outcomes.

A biologically plausible explanation for the performance of FRR is that it integrates information from two complementary pathophysiologic domains. On the one hand, fibrinogen is a classical acute-phase reactant, and increasing evidence suggests that coagulation activation is linked to gout disease activity; for example, Vedder et al. reported higher levels of thrombin generation markers in patients with active gout, supporting a close interplay between inflammation and coagulation in this disease (18). On the other hand, erythrocytes are increasingly recognized as dynamic participants in inflammatory and hemorheological processes rather than passive oxygen carriers alone (19, 20). Studies in rheumatoid arthritis and other inflammatory conditions suggest that RBC-related alterations may be associated with inflammatory burden and adverse clinical outcomes (11, 21–23). Moreover, fibrinogen is a major determinant of erythrocyte aggregation, while inflammation can impair erythrocyte deformability and promote eryptosis, thereby contributing to increased blood viscosity, microcirculatory disturbance, and compromised tissue perfusion (15, 19). Accordingly, FRR may reflect not only the intensity of systemic inflammation but also downstream hemorheological alterations and microvascular dysfunction. This integrated biological signal may help explain why FRR showed broader and stronger associations with disease burden and short-term prognosis than conventional leukocyte-based indices in our cohort.

An interesting finding in our study was that FRR was independently associated with proteinuria, whereas no clear association was observed with serum creatinine or eGFR. One plausible explanation is that FRR may be more sensitive to inflammatory or endothelial injury than to established impairment in glomerular filtration. In CKD populations, albuminuria/proteinuria has been linked to endothelial dysfunction and proinflammatory states (24), and may capture early renal microvascular injury that is not yet fully reflected by creatinine-based filtration measures (25). In the context of hospitalized gout, this pattern suggests that FRR may identify a subgroup with subclinical or early renal involvement despite relatively preserved conventional renal function indices. However, because our study used cross-sectional admission data, this interpretation remains hypothesis-generating and requires validation in prospective studies with quantitative urinary protein assessment.

The association between FRR and hospitalization outcomes should be interpreted with caution. In unadjusted analyses, patients with higher FRR had longer hospital stays, higher hospitalization costs, and a higher rate of 30-day readmission. However, after multivariable adjustment, length of hospital stay and hospitalization cost were not retained in the final linear regression models, whereas high FRR remained independently associated with 30-day readmission. These findings suggest that FRR may be more relevant for identifying patients at increased short-term readmission risk than for independently predicting overall healthcare utilization. This is consistent with prior real-world evidence showing that 30-day readmissions after acute gout hospitalization are common and are associated with worse outcomes and greater healthcare burden (26). In that setting, an inexpensive admission biomarker that helps identify patients at higher short-term risk could be clinically useful. However, this finding should be considered exploratory and requires validation in larger prospective cohorts.

From a practical standpoint, FRR has several potential advantages because it is derived from routine admission laboratory tests and requires no specialized assay. Nevertheless, its clinical utility should be interpreted cautiously. Although FRR showed higher AUCs than NLR and PLR for selected outcomes, its discriminatory ability for tophi and proteinuria was modest, and the readmission analysis was limited by the small number of events and a wide confidence interval. In addition, admission medications, including corticosteroids, colchicine, NSAIDs, and urate-lowering therapy, may have influenced inflammatory markers and clinical outcomes, despite being included as candidate covariates. Therefore, FRR should be regarded as an adjunctive risk-stratification tool rather than a standalone marker of disease severity. Future studies should assess its incremental value beyond established clinical variables, imaging findings, urate measures, and medication exposure.

Several limitations should be acknowledged. First, the retrospective single-center design precludes causal inference and may introduce selection bias and residual confounding. Second, conditions affecting fibrinogen or RBC levels, including active infection, hematologic disorders, malignancy, or other acute inflammatory conditions, could not be completely captured or uniformly excluded. Third, although major covariates and admission medications were adjusted for, medication-related confounding could not be fully eliminated. Fourth, FRR was measured only at admission, and proteinuria was assessed by routine urinalysis rather than quantitative measurement. Finally, the modest discriminatory performance for some outcomes, the small number of readmission events, and the predominance of hospitalized male patients limit the generalizability of the findings.

In conclusion, FRR may be a simple adjunctive biomarker associated with disease burden and inflammatory activity in hospitalized patients with gout, with potential value for identifying selected short-term hospitalization risks that warrants further validation.

## Data Availability

The raw data supporting the conclusions of this article will be made available by the authors, without undue reservation.
